# Supplementary material for: Green Synthesis of Silver Nanoparticles Using Jacobaea maritima and the Evaluation of Their Antibacterial and Anticancer Activities
Source: Int J Mol Sci. 2023 Nov 20;24(22):16512. doi: 10.3390/ijms242216512 (PMC10671674; doi:10.3390/ijms242216512)
Supplement: Supplementary file 1 [file ijms-24-16512-s001.zip › ijms-2691531-supplementary.pdf]

Supplementary Materials Section:

Table S1: Active compounds present in *Jacobaea maritima*

Number	Compound	Exact mass (m/z)
1	protocatechuic acid-O-hexoside	315.0722
2	vanillic acid 4-O-hexoside	329.0878
3	vanillic acid	167.0350
4	protocatechuic acid	153.0193
5	syringic acid 4-O-hexoside	359.0984
6	neochlorogenic acid	353.0878
7	caffeic acid-O-hexoside	341.0878
8	4-hydroxybenzoic acid-O-hexoside	299.0773
9	esculetin-O-hexoside	339.0722
10	caffeic acid-O-hexoside	341.0878
11	quinic acid	191.0561
12	chlorogenic acids	353.0878
13	Quercetin	301.0354
14	4-hydroxybenzoic acid	137.0244
15	caffeic acid-O-hexoside isomer I	341.0878
16	4-caffeoylquinic acid	353.0878
17	p-hydroxyphenylacetic acid	151.0401
18	coumaric acid-O-hexoside	325.0929
19	p-coumaric acid	163.0401
20	caffeic acid	179.0350
21	caffeic acid-O-hexoside isomer II	341.0878
22	5-p-coumaroylquinic acid	337.0929
23	3-hydroxy-dihydrocaffeoyl-5- caffeoylquinic acid	533.1301
24	5-feruoylquinic acid	367.1035
25	m-coumaric acid	163.0401
26	3,4-dicaffeoylquinic acid	515.1195
27	3,5-dicaffeoylquinic acid	515.1195
28	1,5-dicaffeoylquinic acid	515.1195
29	4,5-dicaffeoylquinic acid	515.1195
30	shikimic acid	173.0456
31	3-p-coumaroyl-5- caffeoylquinic acid	499.1246
32	3-caffeoyl-5-p-coumaroylquinic acid	499.1246
33	3-feruoyl-5- caffeoylquinic acid	529.1352
34	3-caffeoyl-5- feruoylquinic acid	529.1352
35	3,4,5-tricaffeoylquinic acid	677.1512
36	6,8-di-C-hexosyl-naringenin	595.1669
37	rutin	609.1461
38	isorhamnetin-O-pentosylhexoside	609.1461
39	isoquercitrin	463.0882
40	kaempferol 7-O-rutinoside	593.1512
41	isorhamnetin 3-O-glucoside	477.1039
42	Quercetin	301.0354
43	kaempferol	285.0405

Figures S1-S8 show absorbance graphs and 96-well plates of all test bacterial strains in this study, which were treated with the extract, AgNPs, and $AgNO_3$. The positive control was the bacterium with media, and the negative control was the media only.

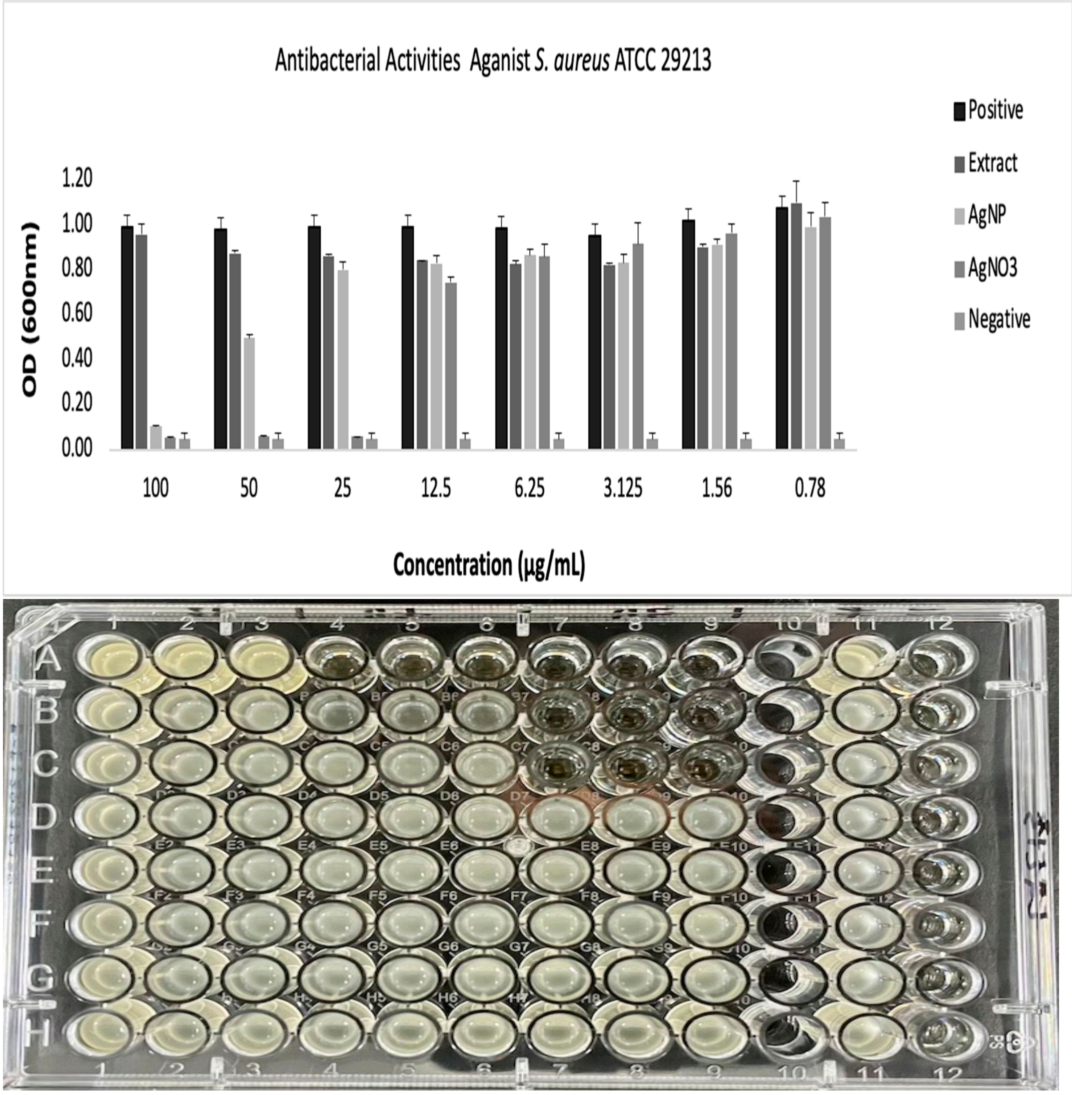

Figure S1: Gram-positive strains, *S. aureus* – ATCC 29213 after treating the bacterium with serially diluted concentrations (100-0.78 µg/mL; from row A to H, respectively) of the *Jacobaea maritima* extract (lines 1-3); AgNPs (lines 4-6); $AgNO_3$ (lines 7-9); positive control (line 11); negative control (line 12).

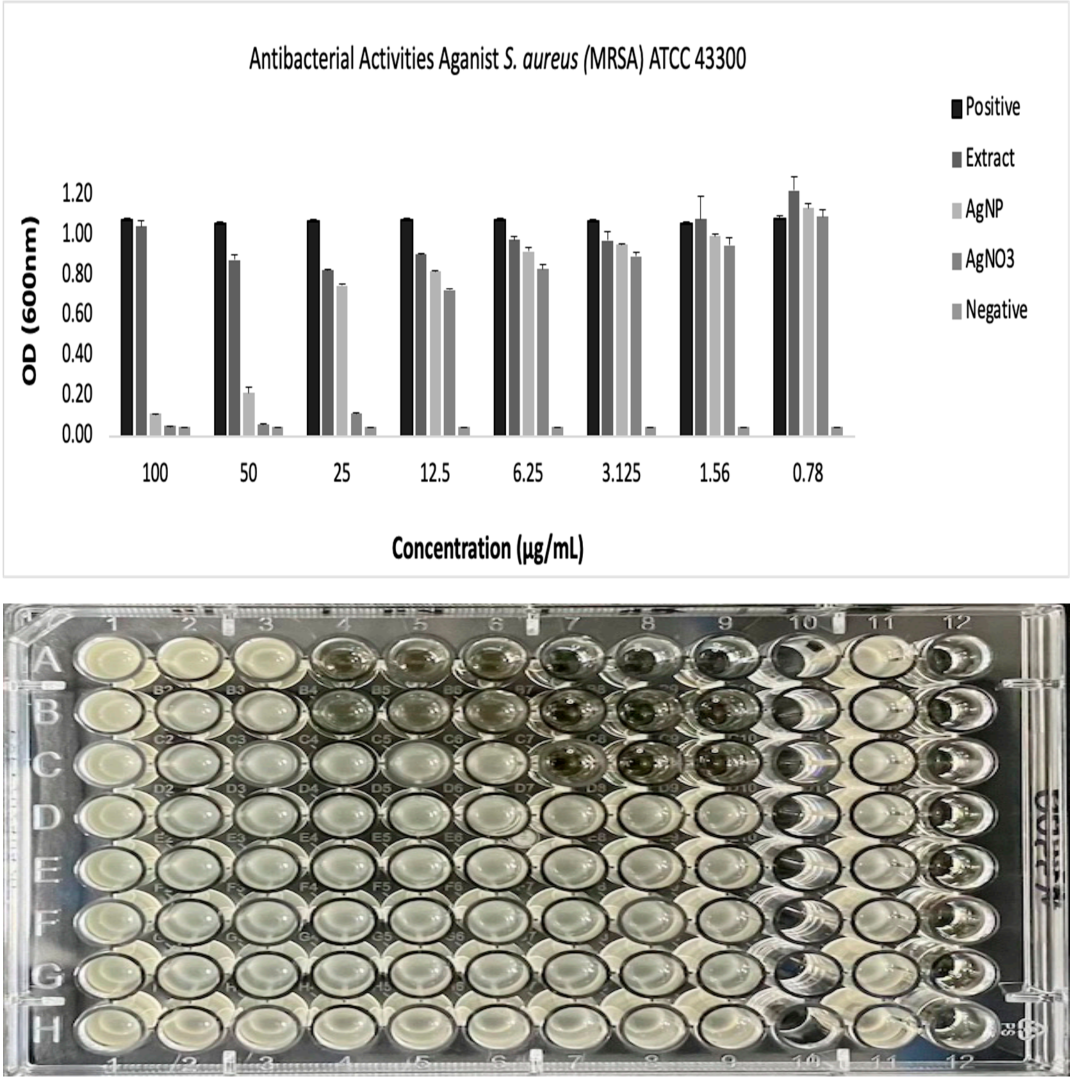

Figure S2: Gram-positive strains, *MRSA* – MDR strain ATCC 43300 after treating the bacterium with serially diluted concentrations (100-0.78 µg/mL; from row A to H, respectively) of the *Jacobaea maritima* extract (lines 1-3); AgNPs (lines 4-6); $AgNO_3$ (lines 7-9); positive control (line 11); negative control (line 12). MDR: multi-drug resistant.

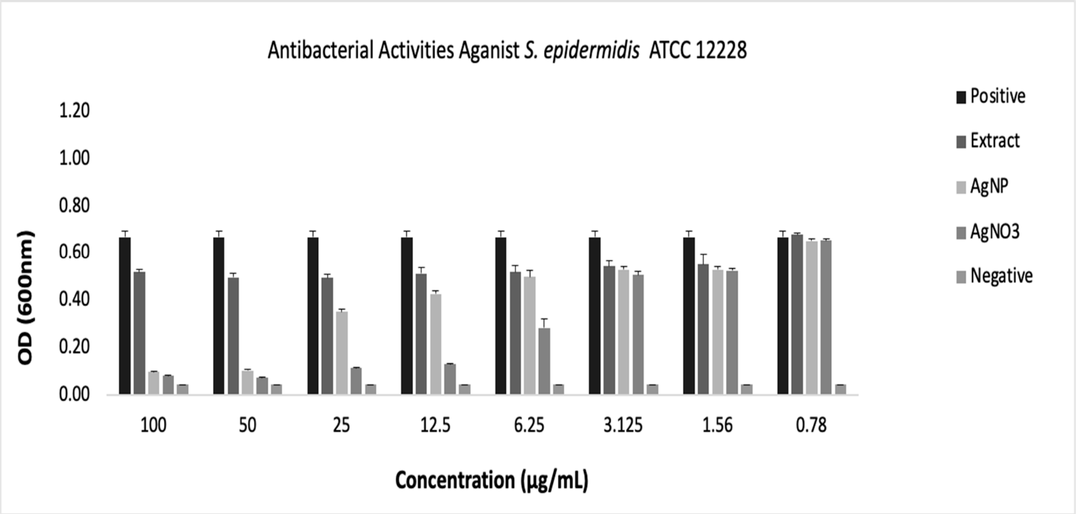

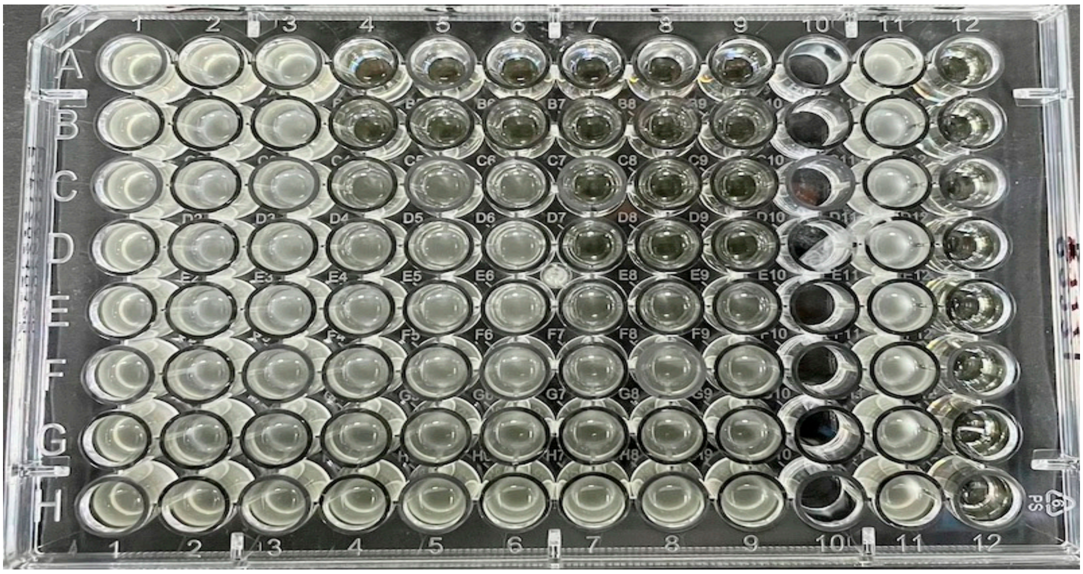

Figure S3: Gram-positive strains, *S. epidermidis* -ATCC 12228 after treating the bacterium with serially diluted concentrations (100-0.78 µg/mL; from row A to H, respectively) of the *Jacobaea maritima* extract (lines 1-3); AgNPs (lines 4-6); $AgNO_3$ (lines 7-9); positive control (line 11); negative control (line 12).

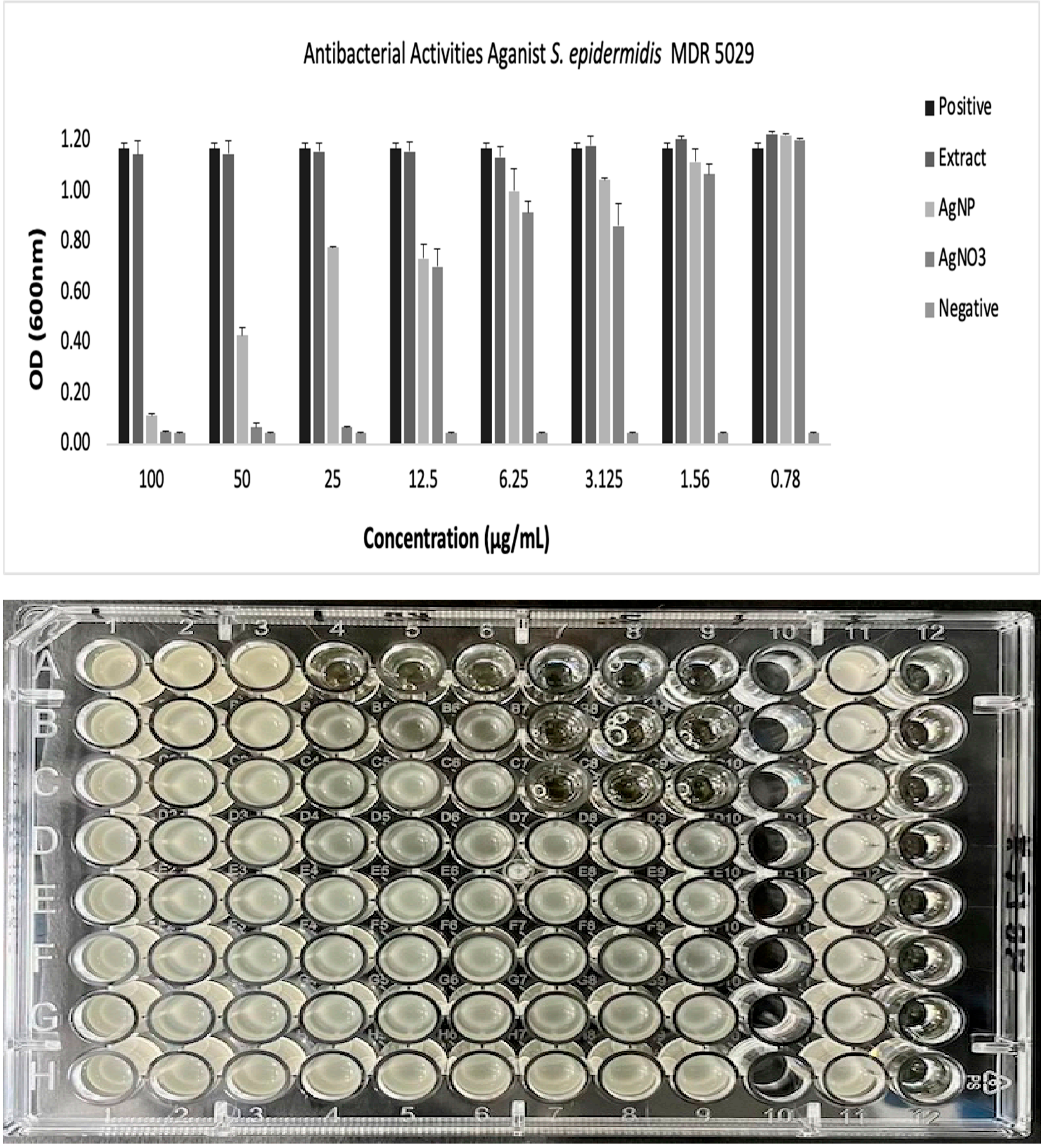

Figure S4: Gram-positive strains, *S. epidermidis* – MDR isolate 5029 after treating the bacterium with serially diluted concentrations (100-0.78 µg/mL; from row A to H, respectively) of the *Jacobaea maritima* extract (lines 1-3); AgNPs (lines 4-6); $AgNO_3$ (lines 7-9); positive control (line 11); negative control (line 12). MDR: multi-drug resistant.

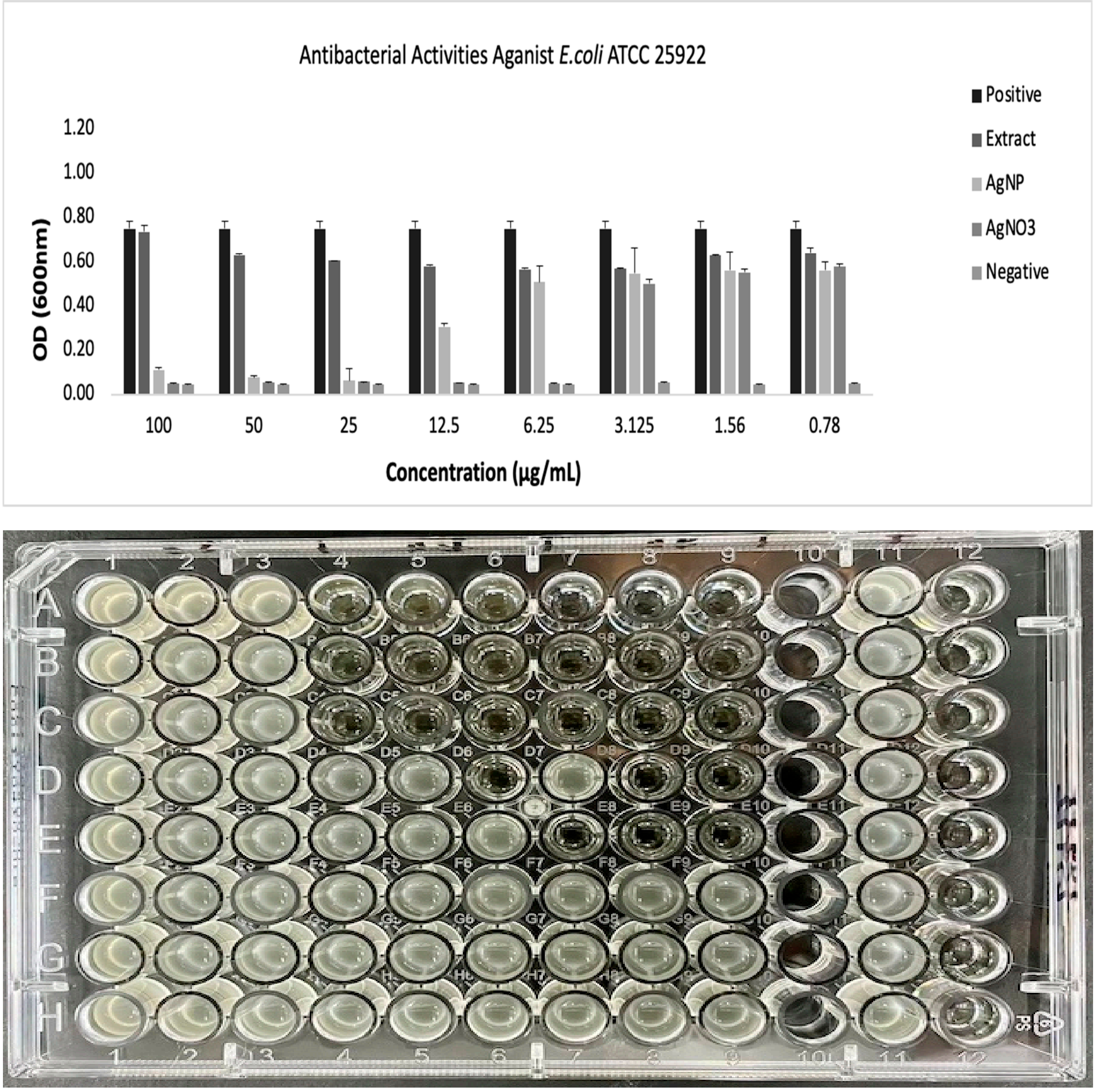

Figure S5: Gram-negative strains, *E. coli*-ATCC 25922 after treating the bacterium with serially diluted concentrations (100-0.78 μg/mL; from row A to H, respectively) of the *Jacobaea maritima* extract (lines 1-3); AgNPs (lines 4-6); $AgNO_3$ (lines 7-9); positive control (line 11); negative control (line 12).

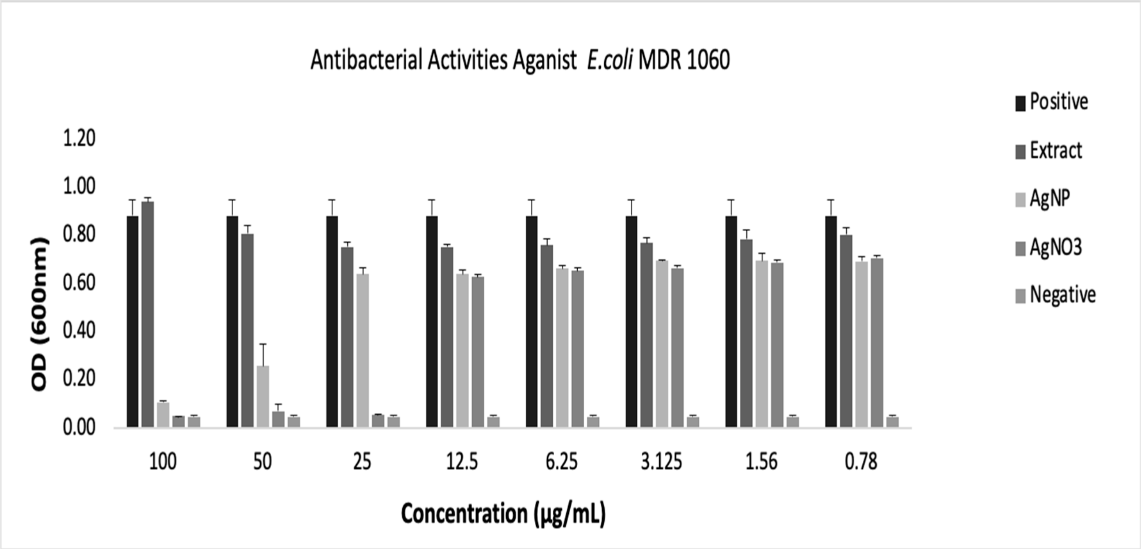

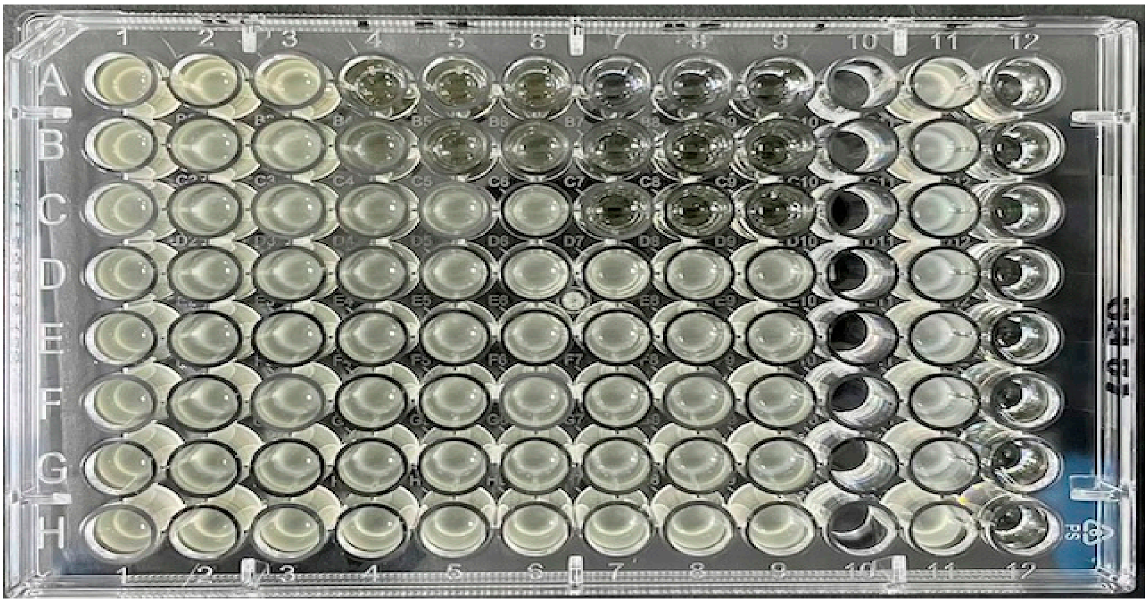

Figure S6: Gram-negative strains, *E. coli* – MDR isolate 1060 after treating the bacterium with serially diluted concentrations (100-0.78 µg/mL; from row A to H, respectively) of the *Jacobaea maritima* extract (lines 1-3); AgNPs (lines 4-6); $AgNO_3$ (lines 7-9); positive control (line 11); negative control (line 12). MDR: multi-drug resistant.

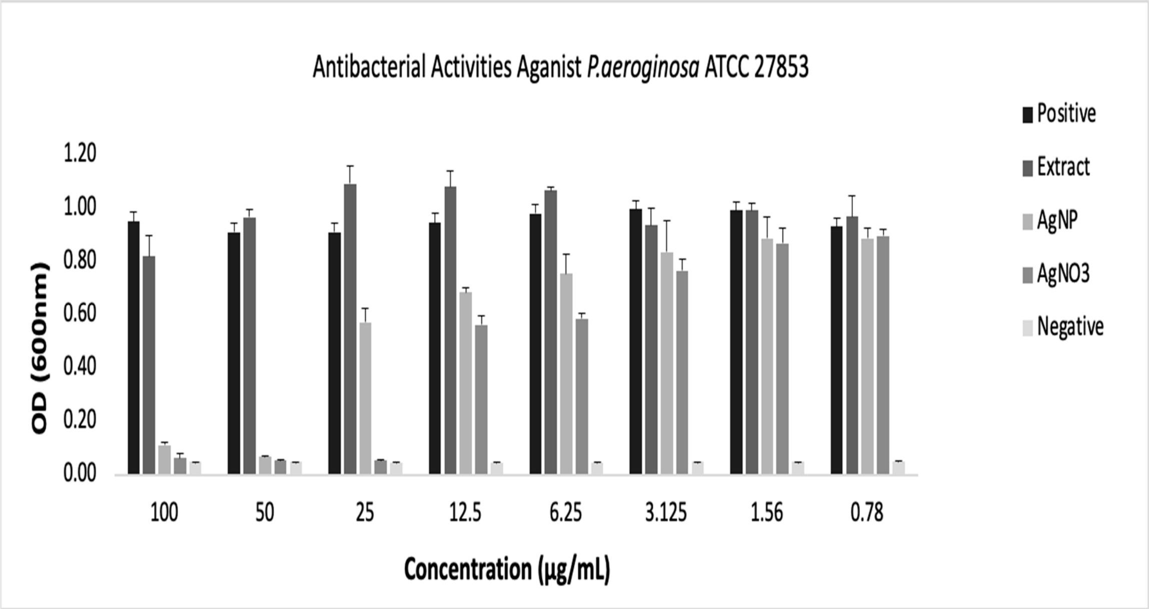

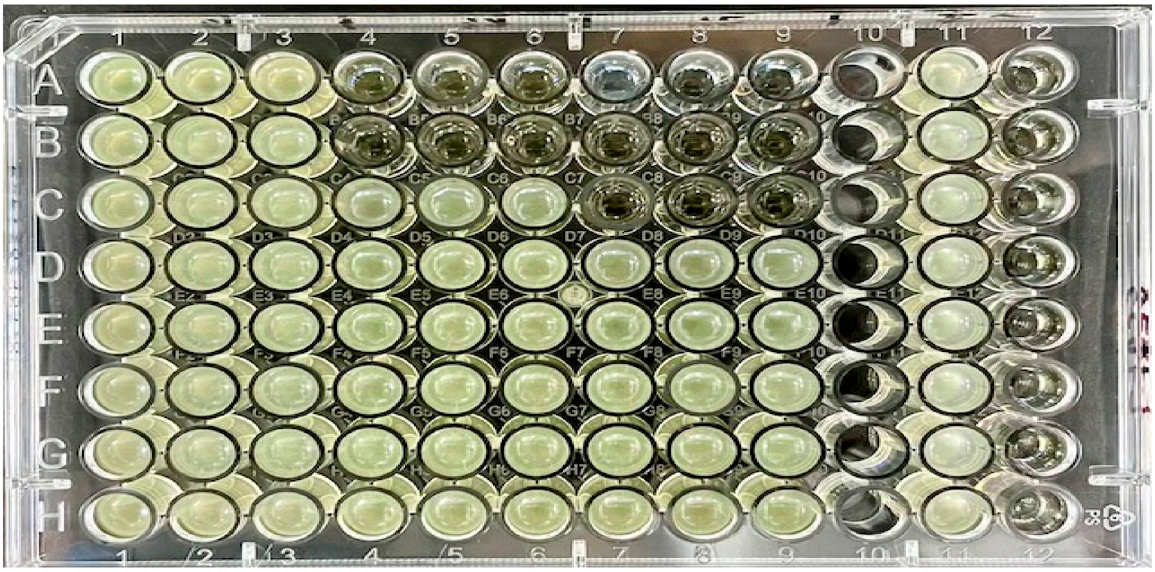

Figure S7: Gram-negative strains, *P. aeruginosa* -ATCC 27853 after treating the bacterium with serially diluted concentrations (100-0.78 µg/mL; from row A to H, respectively) of the *Jacobaea maritima* extract (lines 1-3); AgNPs (lines 4-6); $AgNO_3$ (lines 7-9); positive control (line 11); negative control (line 12).

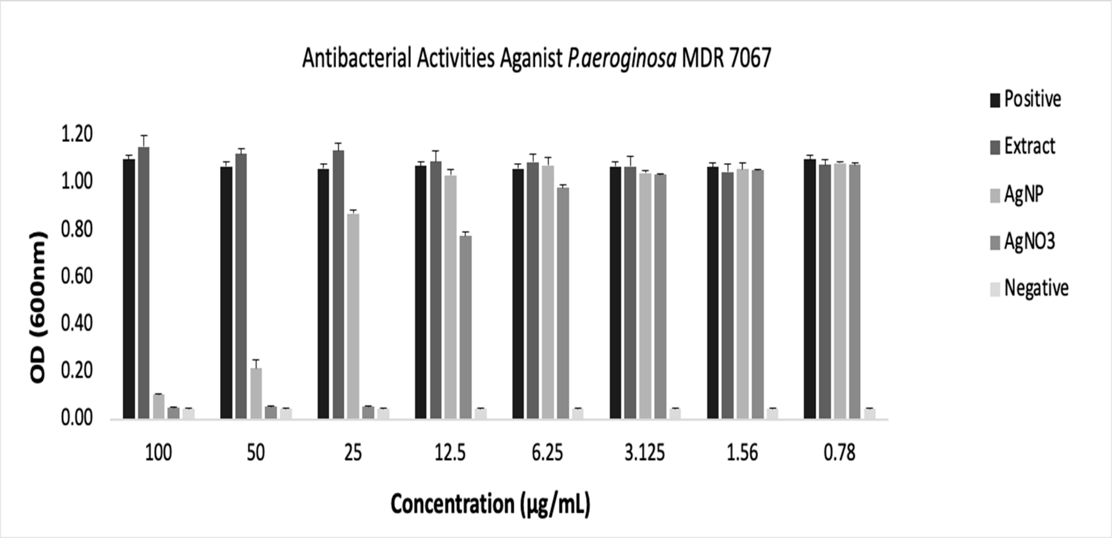

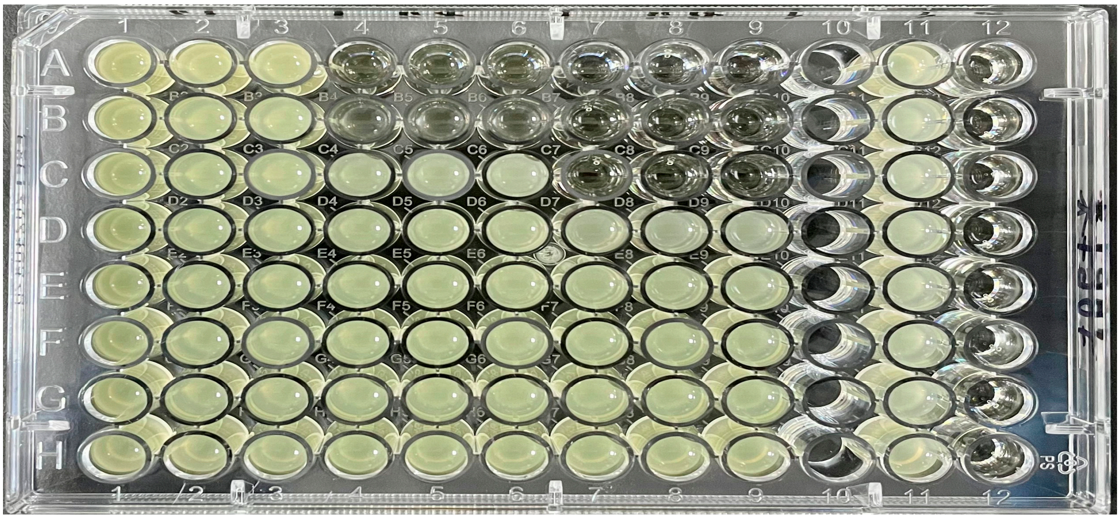

Figure S8: Gram-negative strains, *P. aeruginosa*- MDR isolate 7067 after treating the bacterium with serially diluted concentrations (100-0.78 µg/mL; from row A to H, respectively) of the *Jacobaea maritima* extract (lines 1-3); AgNPs (lines 4-6); $AgNO_3$ (lines 7-9); positive control (line 11); negative control (line 12). MDR: multi-drug resistant.
